# How Do Employers Span the Generations?

**DOI:** 10.1093/geroni/igaa057.2940

**Published:** 2020-12-16

**Authors:** Cort Rudolph

**Affiliations:** Saint Louis University, St Louis, Iowa, United States

## Abstract

The concepts of “generations” and “generational differences” are nearly ubiquitous phenomena. Organizational policy makers often face questions about the purported influence of generations and differences assumed to exist between them for a variety of issues. Given the sheer volume of work dedicated to the topic, both academic and in the popular press, it can be difficult to parse correct from incorrect answers to such questions. In the present article, we pose and answer 10 common questions about generations and generational differences in an attempt to “clear the air” about common thinking, (mis)conceptions, and myths surrounding these phenomena. We offer this work with the goal of clarifying the truth about generations and generational differences, with an eye toward helping organizational policy makers navigate the complexities of these issues and making better decisions about the (assumed) role that generations play across different contexts.

